# Molecular and serological evaluation of zoonotic visceral leishmaniasis in dogs in a rural area of Fars province, southern Iran, as a source of *Leishmania infantum* infection

**DOI:** 10.1002/vms3.432

**Published:** 2021-05-04

**Authors:** Laleh Najafi, Mostafa Omidian, Zahra Rezaei, Saeed Shahabi, Fariba Ghorbani, Nasir Arefkhah, Mehdi Mohebali, Zabiolla Zaraei, Bahador Sarkari

**Affiliations:** ^1^ Department of Parasitology and Mycology School of Medicine Shiraz University of Medical Sciences Shiraz Iran; ^2^ Professor Alborzi Clinical Microbiology Research Center Shiraz University of Medical Sciences Shiraz Iran; ^3^ Department of Parasitology and Mycology School of Medicine Yasuj University of Medical Sciences Yasuj Iran; ^4^ Department of Medical Parasitology and Mycology School of Public Health Tehran University of Medical Sciences Tehran Iran; ^5^ Meshkin‐Shahr Health Station School of Public Health Tehran University of Medical Sciences Tehran Iran; ^6^ Basic Sciences in Infectious Diseases Research Center Shiraz University of Medical Sciences Shiraz Iran

**Keywords:** dogs, Fars province, leishmaniasis, Seroprevalence, southern Iran

## Abstract

Canine visceral leishmaniasis (CVL) is endemic in the southern parts of Iran. The current study aimed at molecular and serological evaluation of zoonotic visceral leishmaniasis in dogs in Fars province, southern Iran. Blood samples were collected from 60 dogs in the three villages in Fars Province. Serum samples were tested for antibodies against *L. infantum* by direct agglutination test (DAT). DNA was extracted from each dog's buffy coat and tested by PCR, targeting the *Leishmania* ITS‐2 region. From a total of 60 studied dogs, 25 (41.7%) were female, and 35 (58.3%) were male. Dogs' age ranged from 1 to 7 years, with a mean age of 2.97 (±1.4) years. Anti‐*Leishmania* antibodies were detected in sera samples of 28 (46.7%) dogs, (titre ≥ 1:320). Out of 28 seropositive cases, 13 (46.4%) were female, and 15 (53.6%) were male. Association between seropositivity and dogs’ clinical signs was statistically significant (*p* < .05). *Leishmania* DNA was detected in the buffy coat of 3 of 60 studied dogs which were all seropositive by DAT. The PCR products were sequenced and molecular analysis showed that two of the isolates were *Leishmania infantum,* and one was *L. tropica*. The high proportion of seropositive dogs indicates the important role of these animals in the epidemiology of the disease in the region. Infected dogs with or without signs, especially those that are molecularly positive, can act as an active reservoir of the disease in the area.

## Graphical abstract

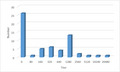

**Impacts**

- Visceral leishmaniasis in 60 dogs in Fars province, southern Iran, was investigated by molecular and serological approaches.
- Anti‐*Leishmania* antibodies were detected in sera of 28 (46.7%) dogs (titre ≥ 1:320), and *Leishmania* DNA was detected in the buffy coat of 3 dogs.
- Molecular analysis showed that two of the *Leishmania* isolates were *Leishmania infantum,* and one was *L. tropica*.

​

​

## 1 INTRODUCTION

Visceral leishmaniasis (VL) is a challenging health problem and one of the most important zoonotic diseases in Iran which often affects children under the age of 5 (Mohebali, 2012; Sarkari et al., 2016). VL is caused by the protozoan parasite of the genus *Leishmania donovani* complex (*donovani, infantum*) that infects humans, dogs and other mammals. During the last decade, the average annual number of VL cases in Iran is reported to be 0.449 cases/100,000 inhabitants (Mohebali, 2013). In addition, a large number of individuals from endemic areas of VL remained asymptomatic despite having the infection (Alborzi et al., 2008). The major cause of VL in Iran is *Leishmania infantum*, although the disease has also been reported due to *Leishmania tropica* (Alborzi et al., 2006; Jafari et al., 2010; Mohebali et al., 2011; Sarkari et al., 2016).

Dogs are considered as the main reservoir host of VL in Iran, while cats (*Felis catus*) may be playing a role in the VL epidemiology in this area (Hatam et al., 2010; Mohebali et al., 2017; Sarkari et al., 2009). Because of the intense cutaneous parasitism of the infected dogs, either symptomatic or asymptomatic, they act as reservoir hosts for VL. Transmission between dogs and humans is via the infected sandflies (Moshfe et al., 2009; Reithinger & Davies, 2002). The prevalence of leishmaniasis in dogs in an area depends on several factors, including weather, the disease endemicity and the method used to detect the infection. Seroprevalence of VL in dogs has been reported from 10% to 37% in endemic areas, whereas molecular studies reported up to 70% infection rates in some areas of the world (Duprey et al., 2006; Fakhar et al., 2011; Haddadzade et al., 2013; Mohebali et al., 2005, 2018). VL in dogs develops as a chronic systemic disease that has a progressive and debilitating pattern. Infection in dogs may occur in the absence of clinical signs, but usually, all dogs with skin manifestations also have visceral involvement. The common signs in VL‐infected dogs are hair loss, lymph node enlargement, spleen enlargement, eye lesions, onychogryphosis, cachexia and nasal bleeding (Noli & Saridomichelakis, 2014). Moreover, asymptomatic dogs could be also highly infectious and have a potential role in maintaining and spreading the parasite in endemic areas (Laurenti et al., 2013).

Different approaches have been utilized for seroepidemiological studies of VL in dogs, among them, DAT is the most common one. On the other hand, molecular methods, based on the detection of parasite‐specific DNA in different tissues, are highly sensitive, specific and reliable methods that allow early detection of the infection, especially in individuals and reservoirs without clinical signs or with hidden infection (Fakhar et al., 2011; Kalayou et al., 2011; Magalhães‐Junior et al., 2014). Of the molecular methods, the use of the ITS‐2 region, which has a different size and nucleotide sequences in different *Leishmania* species, is a very appropriate method for tracing and finding the *Leishmania* species in canine hosts (Schönian et al., 2003).

VL cases have been reported from all 31 provinces of Iran (Mohebali, 2013). However, the disease is mainly endemic in the southern and northwest parts of the country, including Ardebil, East Azerbaijan, Fars and Bushehr provinces. A hospital‐based study in Fars province reported 380 human VL cases during 1999–2014, corresponding to the average hospital admission of 23.75 cases per year (Sarkari, Bavarsad Ahmadpour, et al., 2016). A previous study by Fakhar et al., on 110 dogs from an area in Fars province, has shown that out of 110 dogs, 5.5% (6/110) were seropositive and 23% (25/110) were PCR positive (Fakhar et al., 2012). In Fars province, along with other areas, Sar Mashhad district is known as a human VL‐endemic area where VL cases have been frequently reported in recent decades. It has been reported that about 10% of VL cases in Fars province are actually from this area (Sarkari et al., 2012). In a recent study on children in a rural area in Sar Mashhad district, anti‐*Leishmania* antibodies and *L. infantum* DNA were detected in the serum of 2.8% and 1.3% of the studied children respectively (Layegh Gigloo et al., 2018).

Evaluation of the extent of the disease and detection of *Leishmania* species in reservoirs, including dogs, is a prerequisite phase in the course of surveillance, prevention and control programs in any areas where the disease is present. Since the previous study has shown the important role of dogs in parasite transmission (Cortes et al., 2007; Fakhar et al., 2012) and also most people in rural areas in southern Iran have close contact with dogs, these animals can be potential reservoirs of the VL infection and contribute to the disease transmission. The present study intended to find out the prevalence of VL in dogs in an area of Fars province, southern Iran, as one of the main foci of VL in southern Iran, using the molecular and serological approaches.

## 2 MATERIALS AND METHODS

### 2.1 Study area

**FIGURE 1:**
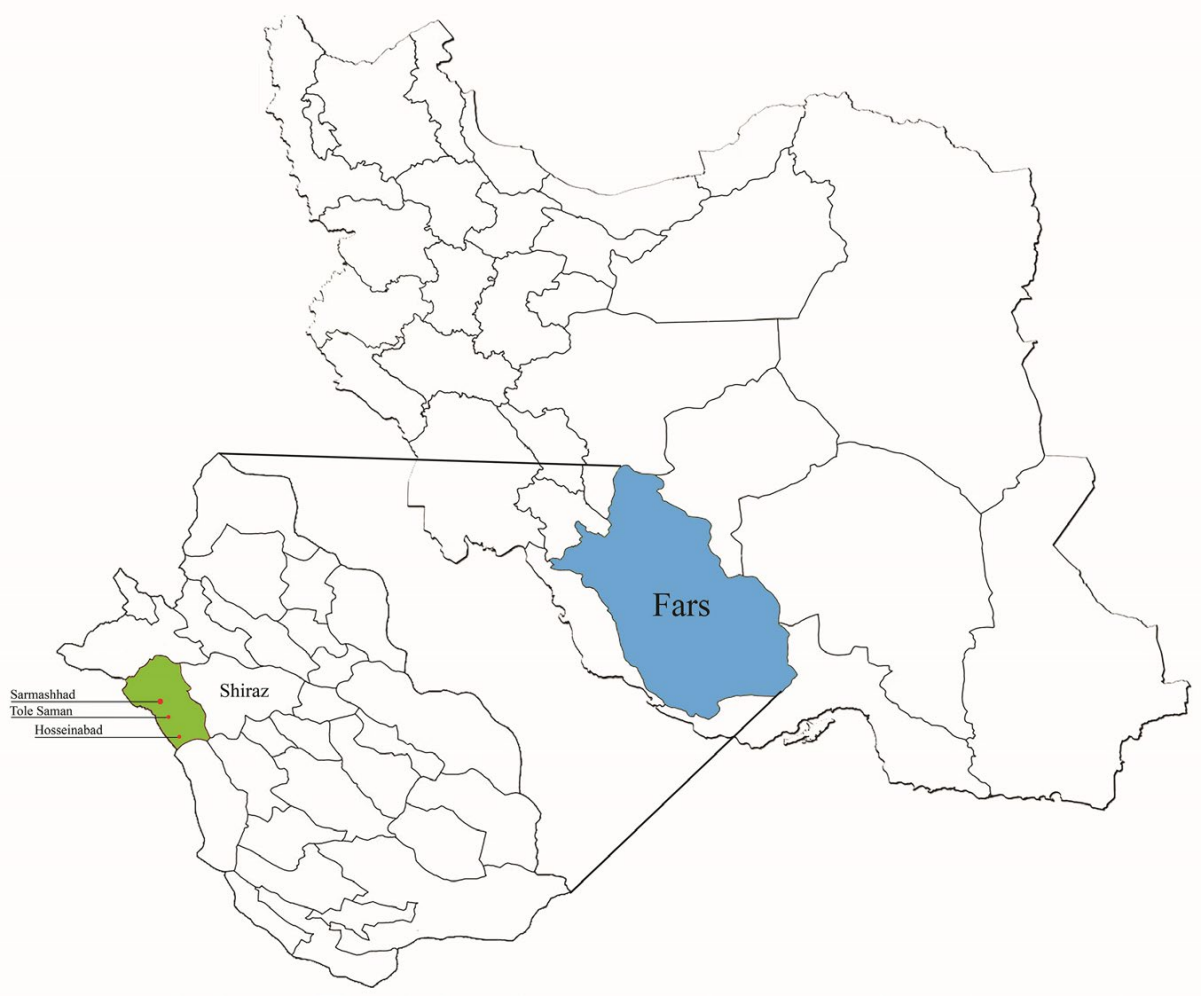
Map of Iran showing the study area

This cross‐sectional descriptive study was carried out in Sar Mashhad district at a 51.701E longitude and 29.294N latitude, in Kazeroun County, in Fars province, southern Iran (Figure 1). The study area includes three villages: Hosseinabad, Sar Mashhad (the most populated one) and Tolesaman. Residences of the area are mostly doing agricultural and animal breeding activities. The region has hot summers and mild winters and is located in the border of Fars and Bushehr provinces.

### 2.2 Sampling

Sampling was done from 60 dogs (both owned and free‐roaming dogs) in June 2018. Sampling has been done in a rural area where owned and free‐roaming dogs are freely roaming around in the village. Owned dogs, like free‐roaming dogs, are kept outdoors and roam freely in the environment. At first, the dogs were evaluated for the presence of any VL clinical signs including hair loss, lymph node or spleen enlargement, eye lesions, onychogryphosis, cachexia and nasal bleeding. Demographic data including the age and sex of the dogs were noted. Then, 10 ml of peripheral blood was collected from the jugular vein by syringe aspiration. Serum and buffy coat were separated and stored at −20°C until use. The sera samples were assessed for anti‐*Leishmania* antibodies by DAT. A nested PCR was used to detect the DNA of *Leishmania* in the dogs’ buffy coat samples.

### 2.3 DAT for the detection of anti‐*Leishmania* antibodies

The *L. infantum* antigens were provided by the parasitology department of the School of Public Health at Tehran University of Medical Sciences. The main procedure for the preparation of DAT antigen was mass cultivation of promastigotes of *L. infantum* LON‐49 in RPMI1640, parasite trypsinization, Coomassie blue staining and fixing with 2% formaldehyde (el Harith et al., 1989). The dog sera samples were evaluated by DAT based on the procedure originally described by Harith et al. (el Harith et al., 1989) and utilized in several studies in Iran (Mohebali et al., 2006; Sarkari et al., 2015). A cut‐off titre of ≥1:320 was considered positive (Mohebali et al., 2005).

### 2.4 DNA extraction and polymerase chain reaction (PCR)

The genomic DNA was extracted from the dogs’ buffy coat, using a manual phenol/chloroform/isoamyl extraction method (Davami et al., 2010). DNA was precipitated in 50 µl of elution buffer and kept at 4°C for subsequent analysis. Nested PCR, targeting the ITS‐2 region of *Leishmania* gene locus (220 bp) was performed, using external F1: 5́ ‐CATGCATGCAGTCGATGCACGTA‐3́ and R1: 5́ ‐ TAGCTAGCTGACGCTAGCTGCC‐3́ and internal F2: 5́ ‐AAT TCA ACT TCG CGT TGG CC‐ 3́ and R2: 5́ ‐CCT CTC TTT TTT CTC TGT GC‐ 3́ primers. PCR was performed at a final volume of 25 µl reaction including extracted DNA (2 µl), primers (1µl from each, 10 p.m.), Taq DNA Polymerase Master Mix (1×, 12.5 µl) and distilled water (9.5 µl). The PCR program was run as follows: pre‐denaturation at 94.5°C for 5 min, denaturation at 94°C for 30 s, annealing at 55˚C for 30 s, extension at 72°C for 30 s and a final extension step at 72°C for 8 min (Layegh Gigloo et al., 2018). Electrophoresis of the PCR products was done on 1.5% agarose gel, followed by visualization by UV trans‐illumination, and photographed. Each run of PCR contained negative and positive control samples including the DNA sample of a healthy individual from a non‐endemic VL area and DNA sample extracted from the *L. infantum* (MCAN/IR/14/M14) promastigote respectively.

### 2.5 Sequencing and species determination

The PCR products were sequenced in both directions in Sanger sequencing‐based methods (provided by the codon genetic group, Tehran, Iran, https://codongeneticgroup.com/) utilized those primers which were used for the PCR. The sequences were compared with the reference sequences in the GenBank, using the BLAST program. Moreover, the ITS‐1 region of the three PCR‐positive dogs was amplified, using a specific primer for the *Leishmania* ITS‐1, (Layegh Gigloo et al., 2018) and sequenced. The sequences were compared with the existing ITS‐1 sequences of some VL patients belongs to the same area.

### 2.6 Statistical analysis

The collected data were analysed by SPSS software (version 18, IBM Inc., USA). The Chi‐square, as well as Fisher's exact test, were used to determine the association between seropositivity to VL and subject variables including age, sex, sampling area and clinical sign.

## 3 RESULTS

**FIGURE 2:**
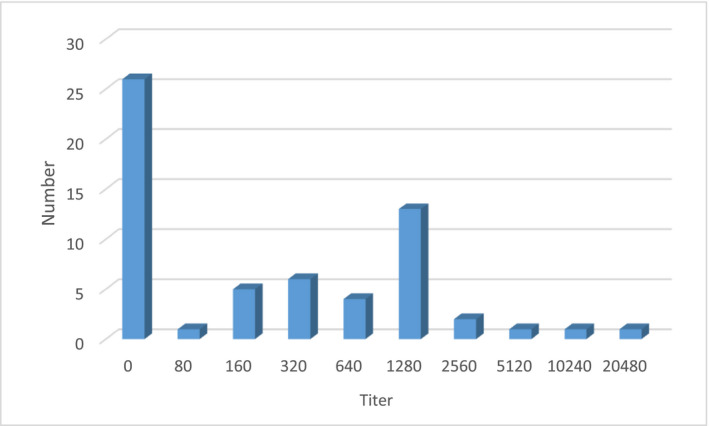
Distribution of anti‐*Leishmania* antibody titres in evaluated dogs by DAT

From a total of 60 studied dogs, 25 (41.7%) were female and 35 (58.3%) were male. The dogs were between 1 and 7 years old with a mean age of 2.97 (±1.4) years. Anti‐*Leishmania* antibodies were detected in sera of 28 (46.7%) of dogs by DAT (titre ≥ 1:320) (Figure 2). The samples were re‐tested to validate the findings. Of the seropositive cases, most (46.4%) were positive with a titre of 1:1,280. Out of 28 seropositive cases, 13 (46.4%) were female, and 15 (53.6%) were male. There was no significant association between seropositivity and dogs’ sex (*p* > .05). Infection was more common (53.6%) in dogs from Sar Mashhad village comparing to the other two villages (Tolesaman and Hosseinabad). A positive association, although not statistically significant, was found between dogs’ age and seropositivity of VL where the rate of infection increased by the animal age. Considering the dogs’ clinical signs, black skin was the most commonly observed sign in the evaluated dogs and there was a significant association between seropositivity to VL and clinical signs of the dogs (*p* < .05). Table 1 shows the characteristics of evaluated dogs and relative seropositivity to *Leishmania*.

**TABLE 1:** Characteristics of evaluated dogs and relative seropositivity to *Leishmania* in free‐roaming dogs in a rural area of Fars province, southern Iran

	Frequency (No.)	Percentage (%)	Positive for anti‐*Leishmania* antibody		*p*‐value
			Frequency (No.)	Percentage (%)	
Sex					
Male	35	58.3	15	42.8	0.60
Female	25	41.7	13	52.0	
Age (Year)^a^					
0–2	16	47.1	6	37.5	0.41
3–4	11	32.3	2	18.2	
5–6	6	17.6	3	50.0	
>6	1	2.9	1	100	
Place					
Sar Mashhad	23	38.3	15	53.6	0.07
Hosseinabad	13	21.7	4	14.3	
Tolesaman	24	40.0	9	32.1	
Clinical signs^b^					
Dermatitis	7	11.7	3	42.8	
Eyeglasses sign^c^	2	3.3	1	50.0	0.04
Scaly, thick or discoloured skin	9	15.0	4	44.4	
Cachexia	7	11.7	4	57.1	

^a^ The age of some dogs was not determined.

^b^ clinical signs were seen in 25 cases.

^c^ Keratoconjunctivitis: The dog looks like she wears glasses.

**FIGURE 3:**
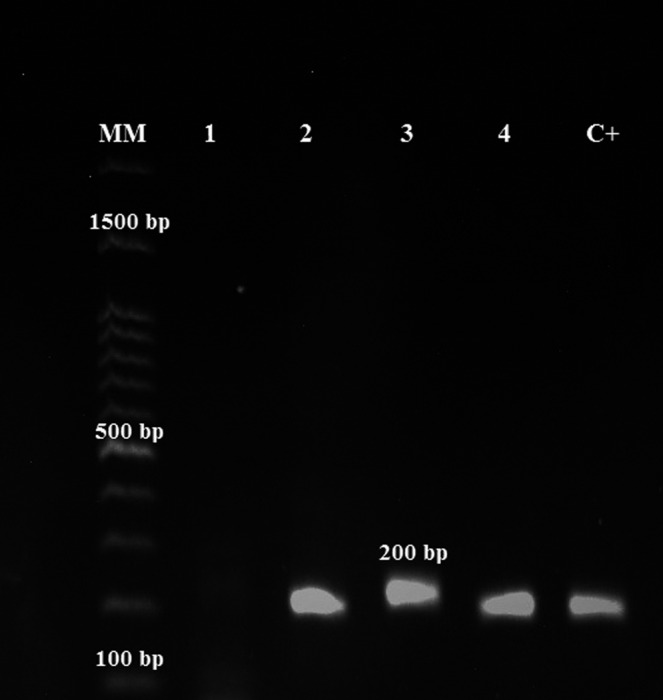
PCR products electrophoresis of ITS‐2 of *Leishmania infantum* on an agarose gel. MM: 100‐bp molecular marker; lane 1: negative control, lanes 2–4: *Leishmania* spp isolates in the present study; C+: positive control

**FIGURE 4:**
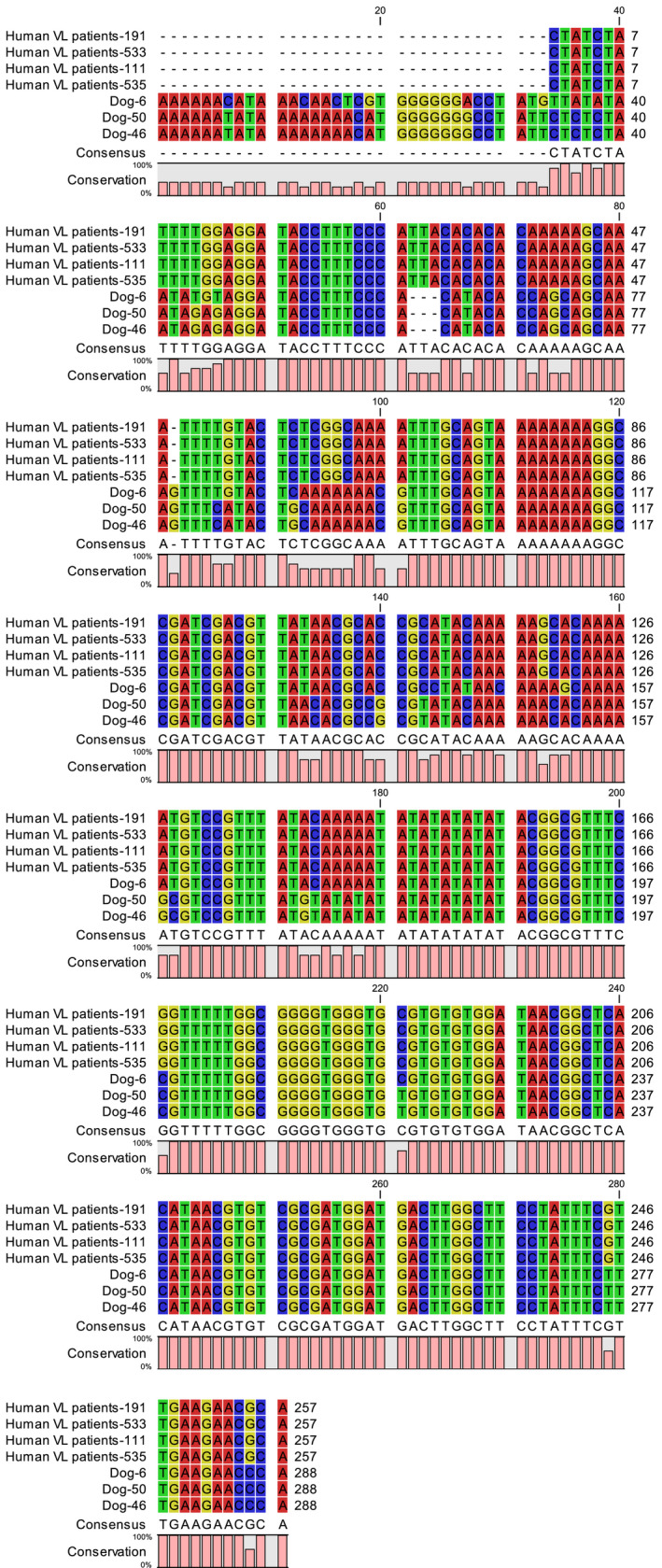
alignment of sequences of ITS‐1 region of *Leishmania* isolated from dogs (in the current study) with existing ITS‐1 sequences of some VL patients belongs to the same area

*Leishmania* DNA was detected in the buffy coat of three dogs, which were all DAT positive (Figure 3). Two PCR‐positive dogs had clinical signs in favour of VL. Molecular analysis revealed that two of the isolates were *L. infantum,* and one was *L. tropica*. The sequences of the ITS‐1 region of the three dog samples were compared with the existing ITS‐1 sequences of VL patients. The results showed more than 96% similarity between ITS‐1 sequences of *Leishmania* isolated from dogs and humans (Figure 4).

## 4 DISCUSSION

VL infection may be asymptomatic in dogs and may persist for the dog's lifetime without clinical signs, whereas some of the infected dogs may present clinical manifestations of the disease for many years or throughout their life. It has been documented that about half of the infected dogs may have no clinical signs and like symptomatic dogs, play an active role in transmitting the *L. infantum* to susceptible humans or animals (Aoun et al., 2009; Mohebali et al., 2005). Due to the large population of dogs in Iran, which is estimated to be seven dogs per 100 people in some of the VL‐endemic areas, and close contact of humans, especially children, with dogs, they have been considered as the most important reservoir of VL for human infection (Mohebali, 2012; Mohebali et al., 2005).

In the current study, the *Leishmania* infection in dogs in a rural area in Kazeroun County, as a focus of VL in Fars province was evaluated. High seroprevalence of VL (46.7%) was observed for *Leishmania* infection in the evaluated dogs. Based on a recent systematic review and meta‐analysis, the overall prevalence of canine VL in Iran was estimated to be 16% (Shokri et al., 2017). This rate of infection is said to be higher in rural (36%) than urban dogs (Shokri et al., 2017).

In a serosurvey of canine VL in Meshkin‐Shahr district, northwest of Iran, 17.4% of the studied dogs were found to be seropositive for *Leishmania* by DAT (Moshfe et al., 2008). Given that our study was conducted on rural dogs in an endemic area of VL, the findings are consistent with those of previous studies on VL infection in rural dogs (Shokri et al., 2017).

In the present study, PCR has been positive in only 8.9% of DAT‐positive samples. This low prevalence of *Leishmania* by PCR may be linked to the low sensitivity of this method in the detection of asymptomatic cases since most of the dogs in our study had no sign of VL. On the other hand, the sensitivity and specificity of PCR depend on the target gene, DNA extraction method and source of biopsy. As bone marrow, spleen or lymph node biopsies are difficult to attain and need an invasive method to obtain the samples, blood sampling is often used for DNA extraction and molecular testing. Usually, parasite load in blood samples compared to spleen, bone marrow and lymph node samples are low and there may be PCR inhibitors in blood samples, which may affect the sensitivity of the PCR method (Lachaud et al., 2001; Reithinger et al., 2000).

Several previous studies demonstrated a significant association between *Leishmania* infection rate in dogs and the animal age (Mohebali et al., 2005; Moshfe et al., 2009; Sousa et al., 2011). The findings of the present study also showed a positive association, although not statistically significant, between dogs’ age and seropositivity to VL where the rate of infection increased by the animal age.

In the current study, contrary to a previous study, no significant association was seen between VL seropositivity and the dog's sex. Such observations have been reported in some of the previous studies (Mohebali et al., 2005; Sousa et al., 2011).

In the present study, the species of *Leishmania* in one of the dogs was found to be *L. tropica*. The etiological agent of canine VL is usually *L. infantum*, yet *L. tropica* has infrequently been reported as the causative agent of canine VL (Guessous‐Idrissi et al., 1997; Hajaran et al., 2007). Such cases have been reported from the northwest of Iran where human VL is endemic (Hajaran et al., 2007). Moreover, human VL due to *L. tropica* has been reported from southern Iran (Alborzi et al., 2006; Sarkari, Naraki, et al., 2016) and in American soldiers recruited in Iraq (Crum et al., 2005). It is worth mentioning that sera from dogs infected with *L. tropica* are reactive with *L. infantum* antigen, and that is why the dogs infected with *L. tropica* in our study have been seropositive by DAT, which uses *L. infantum* promastigotes (Baneth et al., 2017). As dog culling is a relatively ineffective control method for VL, other control approaches such as identifying the infected dogs and treating them, as well as interventions directed at the sandfly vector, can be considered in controlling the disease. The main limitation of this study is the relatively small number of samples studied.

## 5 CONCLUSION

The high proportion of seropositive dogs indicates the important role of these animals in the epidemiology of the disease in the area where human VL had been previously identified. Infected dogs with or without signs, especially those that are molecularly positive, can act as an active reservoir of disease in the area. Findings of the present study can be used in the control as well as the surveillance of human and canine VL in this area or areas with similar settings.

## CONFLICT OF INTEREST

The authors declare that they have no competing interests.

## AUTHOR CONTRIBUTIONS

**Laleh Najafi:** Data curation; Formal analysis; Investigation; Methodology; Writing‐original draft. **Mostafa Omidian:** Data curation; Methodology; Writing‐original draft. **Zahra Rezaei:** Data curation; Formal analysis; Investigation; Methodology; Writing‐review & editing. **Saeed Shahabi:** Data curation; Formal analysis; Methodology; Software; Writing‐original draft. **Fariba Ghorbani:** Data curation; Investigation; Methodology. **Nasir Arefkhah:** Data curation; Methodology; Writing‐original draft. **Mehdi Mohebali:** Conceptualization; Supervision; Validation; Writing‐review & editing. **Zabiolla Zaraei:** Data curation; Methodology; Writing‐original draft. **Bahador Sarkari:** Conceptualization; Formal analysis; Methodology; Project administration; Supervision; Writing‐review & editing.

### PEER REVIEW

The peer review history for this article is available at https://publons.com/publon/10.1002/vms3.432.
